# Development of Novel Peptides That Target the Ninjurin 1 and 2 Pathways to Inhibit Cell Growth and Survival via p53

**DOI:** 10.3390/cells14060401

**Published:** 2025-03-09

**Authors:** Jin Zhang, Xiangmudong Kong, Xinbin Chen

**Affiliations:** Comparative Oncology Laboratory, Schools of Veterinary Medicine and Medicine, University of California, Davis, CA 95616, USA; dkkong@ucdavis.edu

**Keywords:** Ninjurin 1, Ninjuring 2, peptide, p53, cancer treatment

## Abstract

Ninjurin 1 and 2 (NINJ1, NINJ2) belong to the homophilic cell adhesion family and play significant roles in cellular communication and tissue development. While both NINJ1 and NINJ2 are found to be over-expressed in several types of cancers, it remains unclear whether they can be targeted for cancer treatment. In this study, we aimed to develop NINJ1/2 peptides derived from the N-terminal extracellular domain that can elicit growth suppression and thus possess therapeutic potentials. We found that peptide NINJ1-A, which is derived from the N-terminal adhesion motif of NINJ1, was able to inhibit cell growth in a NINJ1- or p53-dependent manner. Similarly, peptide NINJ2-A, which is derived from the N-terminal adhesion motif of NINJ2, was able to inhibit cell growth in a NINJ2- or p53-dependent manner. We also found that NINJ1 and NINJ2 physically interact via their respective N-terminal domains. Interestingly, NINJ1-B and NINJ2-B peptides, which were derived from the N-terminal amphipathic helix domains of NINJ1 and NINJ2, respectively, were able to disrupt NINJ1-NINJ2 interaction and inhibit cell growth in a NINJ1/NINJ2-dependent manner. Notably, NINJ1-B and NINJ2-B peptides demonstrated greater potency in growth suppression than NINJ1-A and NINJ2-A peptides, respectively. Mechanistically, we found that NINJ1-B and NINJ2-B peptides were able to induce p53 expression and suppress cell growth in a p53-dependent manner. Together, our findings provide valuable insights into the development of NINJ1/NINJ2 peptides as potential cancer therapeutics, particularly for cancers harboring wild-type p53.

## 1. Introduction

Cell adhesion molecules (CAMs) are proteins located on the cell surface and extracellular matrices (ECMs) and involved in homophilic and heterophilic protein–protein interactions during the cell adhesion process [1,2]. Homophilic interactions represent interactions between two of the same proteins while heterophilic interactions between two of different cell adhesion molecules. CAMs typically consist of three domains: an extracellular domain, a transmembrane domain, and an intracellular domain [3]. The extracellular domain mediates cell adhesion while the intracellular domain connects to the cytoskeleton, either directly or through scaffolding proteins, and facilitates intracellular signaling. The transmembrane domains are hydrophobic in nature and embedded in the plasma membrane to prevent the CAM from free-floating. CAMs are essential for maintaining tissue architecture and cellular communication and play vital roles in various cellular processes, including cellular growth and differentiation, migration and invasion, immune responses, and cancer progression and metastasis [4,5,6]. Since CAMs are frequently altered in cancers, contributing to tumor growth and metastasis, targeting CAMs has emerged as a promising strategy for cancer therapy [7,8,9].

The Nerve Injury-induced Protein 1 and 2 (Ninjurin1 and 2, or NINJ1, and NINJ2) were originally found to be significantly upregulated in response to nerve damage in Schwann cells and dorsal root ganglion neurons, facilitating nerve regeneration by promoting neurite extension [10,11]. NINJ1 and NINJ2 are structurally characterized as two-pass transmembrane proteins with an extracellular domain that functions as homophilic adhesion molecules. NINJ1 and NINJ2 are implicated in multiple processes, such as cell adhesion, migration, and intercellular communication. Ninjurin 1 is expressed in a broader range of tissues, including immune cells and various epithelial cells, whereas Ninjurin 2 expression is restricted to immune cell lineages and certain neuronal populations [12]. As both Ninjurin 1 and 2 are expressed on the cell surface of various immune cells, including macrophages, neutrophils, and endothelial cells, it was suggested that NINJ1/2 are involved in the recruitment of immune cells to the site of injury [13]. In addition to cell adhesion, recent studies showed that the N-terminus of NINJ1 aggregates and promotes lytic membrane rupture and subsequently, several types of cell death, such as pyroptosis and ferroptosis [14,15,16,17]. Interestingly, we found that NINJ2 prevents pyroptosis by modulating NLRP3 and p53 expression [18], possibly due to its structural difference from NINJ1 [14]. These data suggest that NINJ1 and NINJ2 share a substantial overlap in function, but in some cases, they may exert distinct functions due to the expression pattern or structure differences.

Our previous studies showed that NINJ1 and NINJ2 are a targets of p53 and in turn regulate p53 expression [18,19,20,21]. We also found a peptide derived from the N-terminal adhesion domain of NINJ1 inhibits cancer cell growth in a p53-dependent manner [19] Interestingly, when searching for the TCGA database, we found that both NINJ1 and NINJ2 are found to be over-expressed in several types of cancer, such as breast cancer, liver cancer and head–neck squamous cell carcinoma. These observations let us postulate whether NINJ1 and NINJ2 can be targeted for cancer therapy. To this end, several peptides spanning the N-terminal extracellular domain of NINJ1 or NINJ2 were synthesized and tested for their ability to kill tumor cells. We identified two peptides derived from the amphipathic helix of NINJ1 and NINJ2, called NINJ1-B and NINJ2-B, respectively, were able to disrupt the interaction between NINJ1 and NINJ2 and inhibit cancer cell growth in NINJ1/2- and p53-dependent manners. Thus, our study has laid a foundation for developing peptide-based strategies to target NINJ1 and NINJ2 for cancer therapy.

## 2. Material and Methods

### 2.1. Reagents

Anti-Actin (sc-47778) and anti-p53 (sc-126) were purchased from Santa Cruz Biotechnology (Dallas, TX, USA). Anti-NINJ1 (ab213695) was purchased from Abcam (Cambridge, UK). Anti-HA (923502) was purchased from Biolegend (San Diego, CA, USA). Anti-Flag (F3165) was purchased from Millipore Sigma (St. Louis, MO, USA). Anti-NINJ2 (14085-1-AP) was purchased from Proteintech (Rosemont, IL, USA). The WesternBright Sirius HRP substrate (Cat# K12043-D20) was purchased from Advansta (San Jose, CA, USA). JetPRIME transfection reagent was purchased from Polyplus (Illkirch, France). Doxorubicin was purchased from Fisher Scientific (Waltham, MA, USA). LPS and Nigericin were purchased from Cell Signaling (Danvers, MA, USA).

### 2.2. Cell Culture

MCF7, Molt4 and 293T cells were purchased from the American Type Culture Collection (ATCC) (Manassas, VA, USA). MCF7 cells, NINJ2-KO MCF7 and Mol4 Cells, and p53-KO MCF7 cells were generated previously [18,19,21,22]. All the cells used in the study contain wild-type p53. All the cells were used below passage 25 or within 2 months after thawing. Since all cell lines from ATCC have been thoroughly tested and authenticated, we did not authenticate the cell lines used in this study. All the cells and their derivatives were cultured in Dulbecco’s modified Eagle’s medium (Life Technologies, Waltham, MA, USA) supplemented with 10% fetal bovine serum (Life Technologies).

### 2.3. Plasmids

pcDNA3 vector expressing HA-tagged NINJ1 was generated previously [21]. To generate pcDNA3 vector expressing Flag-tagged NINJ2, a cDNA clone (Clone ID: 5300041) expressing full-length Human NINJ2 gene was purchased from Thermo Fisher (Waltham, MA, USA). This cDNA clone was then used as a template for PCR reaction with a forward primer, 5′-GGG GGA ATT CGC CAC CAT GGA ATC AGC AAG AGA AAA C-3′ and a reverse primer, 5′-GGG GCT CGA GCT ACT TGT CGT CAT CGT CTT TGT AGT CCT GCT GGG GTG CCA TGT CCA T-3′. The resulting PCR product was cloned into pcDNA3 vector via EcoRI and XhoI to generate pcDNA3-NINJ2-Flag vector. To generate pcDNA3-NINJ2-HA, the same method was used except that the reverse primer used was 5′-GGG GCT CGA GTC AAG CGT AAT CTG GAA CAT CGT ATG GGT AGA GAG GAT TCC TTG AGG C-3′.

To generate pcDNA3 vector expressing Flag-tagged N-terminal NINJ1 deletion mutant, PCR was performed using pcDNA3-NINJ1 plasmid as a template along with a forward primer, 5′-GGG GGA ATT CGC CAC CAT GGT GGT CCT CAT CTC CAT CTC-3′, and a reverse primer, 5′-GAC TCG AGC TAC TTG TCG TCG TCG TCC TTG TAG TCG ATG TCG TGG TCC TTG TAG TCA CCG TCG TGG TCC TTG TAG TCC TGC TGG GGT GCC ATG TCC AT-3′. The PCR product was then cloned into pcDNA3 via EcoRI and XhoI and the resulting plasmid was named pcDNA3-Flag-NINJ1-∆N. To generate pcDNA3 vector expressing Flag-tagged C-terminal NINJ1 deletion mutant, the same method was used except that the primers used were a forward primer, 5′-GGG GGA ATT CGC CAC CAT GGA CTC GGG AAC CGA GGA G-3′, and a reverse primer, 5′-GAC TCG AGC TAC TTG TCG TCG TCG TCC TTG TAG TCG ATG TCG TGG TCC TTG TAG TCA CCG TCG TGG TCC TTG TAG TCG ACC CCG AAG GCC GTG ATG A-3′. The resulting plasmid is named pcDNA3-Flag-NINJ1-∆C.

To generate pcDNA3 vector expressing Flag-tagged N-terminal NINJ2 deletion mutant, PCR was performed using pcDNA3-NINJ2 plasmid as a template along with a forward primer, 5′-GGG GGA ATT CGC CAC CAT GCT GGT CAC CCT CAT CAG CCT-3′, and a reverse primer, 5′-GAC TCG AGT CAC TTG TCG TCG TCG TCC TTG TAG TCG ATG TCG TGG TCC TTG TAG TCA CCG TCG TGG TCC TTG TAG TCG AGA GGA TTC CTT GAG GCC CT-3′. The PCR product was then cloned into pcDNA3 via EcoRI and XhoI and the resulting plasmid was named pcDNA3-Flag-NINJ2-∆N. To generate pcDNA3 vector expressing Flag-tagged C-terminal NINJ2 deletion mutant, the same method was used except the primers were a forward primer, 5′-GGG GGA ATT CGC CAC CAT GGA ATC AGC AAG AGA AAA C-3′ and a reverse primer, 5′-GAC TCG AGT CAC TTG TCG TCG TCG TCC TTG TAG TCG ATG TCG TGG TCC TTG TAG TCA CCG TCG TGG TCC TTG TAG TCT GCC CCG AAG GCT GTA ATG A-3′. The resulting plasmid was named pcDNA3-Flag-NINJ2-∆C.

### 2.4. Cell Viability Assay

This assay was performed using a CellTiter-Glo assay kit (Promega, Madison, WI, USA) according to the manufacturer’s instructions. Briefly, 5 × 10^3^ cells in 100 μL were seeded per well in a 96-well plate overnight. The next day, the cells were subjected to various treatments for 24 or 48 h. At the end of treatment, 100 μL of CellTiter-Glo reagent was added to each well and incubated for 10 min at room temperature. The luminescence was recorded in the spectraMAX Gemini microplate reader (Molecular Device, Silicon Valley, CA, USA).

### 2.5. LDH Release Assay

This assay was performed using LDH-Glo™ Cytotoxicity Assay (Promega) according to the manufacturer’s instructions. Overnight, 5 × 10^3^ cells were suspended in 100 μL culture medium and seeded in a well of 96-well plate, followed by various treatments. At the end of treatment, 50 uL of culture medium was removed and then mixed with an equal amount of LDH detection reagent for 60 min at room temperature before recording luminescence. The relative % of LDH release is calculated by using the formula: (Experimental LDH Release − Medium Background)/(Maximum LDH Release Control − Medium Background).

### 2.6. Immunoprecipitation and Western Blot Analysis

For the immunoprecipitation assay, cells were lysed in NP-40 lysis buffer (50 mM Tris HCl, pH 7.4, 150 mM NaCl, 1 mM EDTA, and 1% NP-40) supplemented with the proteinase inhibitor cocktail (100 μg/mL). Cell lysates were then incubated with 1 μg of control IgG or antibody along with magnetic protein A/G beads overnight at 4 °C. The immunocomplex was subjected to Western blot analysis. For Western blot analysis, this assay was performed as described previously [23]. In brief, whole-cell lysates were separated on 10–13% SDS-polyacrylamide gels and transferred to nitrocellulose membranes. The membranes were incubated with primary and secondary antibodies at the indicated time. To visualize the protein band, the membrane was incubated with the WesternBright Sirius HRP substrate (Advansta, San Jose, CA, USA), followed by detection using the UVP ChemStudio with VisionWorks LS software 8.2 (Analytik Jena, Jena, Germany).

### 2.7. Colony Formation Assay

Two thousand cells were seeded per well in a six-well plate and the medium was replenished every 3 days for two weeks. Once visible colonies formed, the cells were fixed with methanol/glacial acetic acid (7:1 volume) for 20 min at room temperature and washed three times with distilled water. Colonies were then stained with 0.1% crystal violet for 20 min at room temperature, washed three times with distilled water, and air-dried.

### 2.8. Peptide Synthesis

Control peptide (STLWDTAELWQ), NINJ1-A peptide, NINJ1-B peptide, NINJ2-A peptide and NINJ2-B peptide were synthesized by GenScript (Piscataway, NJ, USA) with purity ≥ 96.4%, as determined by mass spectrometry. Each peptide was dissolved in distilled water.

### 2.9. Statistical Analysis

Student’s *t* test was used for statistical analysis. *p* < 0.05 is considered as significant.

## 3. Results

### 3.1. NINJ2-A Peptide, Which Is Derived from the N-Terminal Adhesion Motif of NINJ2, Inhibits Cell Growth in a NINJ2-Dependent Manner

**Figure 1:**
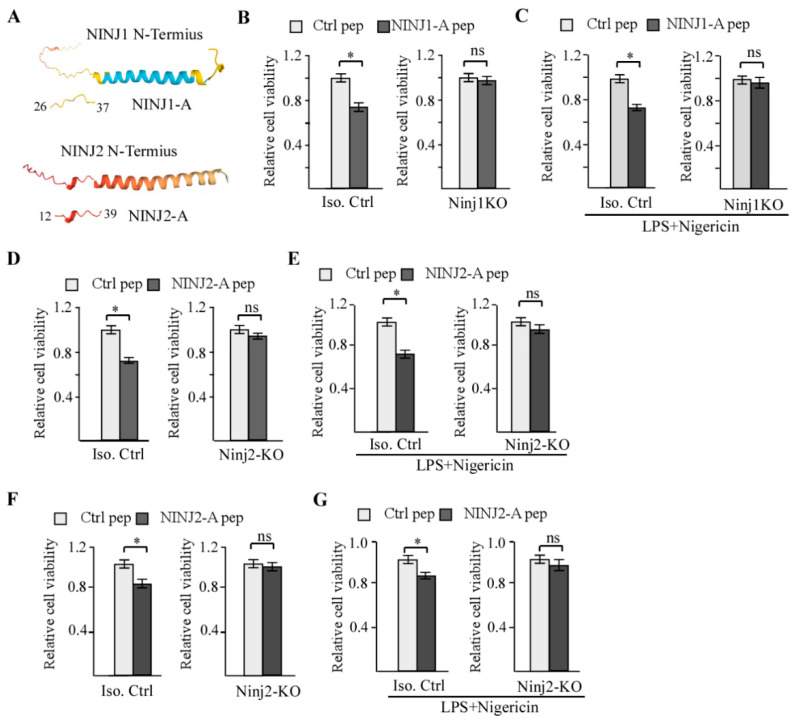
Peptides derived from N-terminus of NINJ1 and NINJ2 proteins inhibit cell growth (**A**) Schematic representation of the N-termini of NINJ1 and NINJ2 proteins and the location of NINJ1-A and NINJ2-A peptides. (**B**,**C**) Isogenic control (**C**) and NINJ1-KO MCF7 cells were treated with 20 μM of control peptide or NINJ1-A peptide for 24 h, and followed by mock-treatment or treatment with LPS (0.5 μg/mL) and Nigericin (10 μM) for 24 h. The cell viability was measured by CellTiter-Glo assay. * indicated *p* < 0.05 by student’s test; n.s, indicated no significance. (**D**,**E**) Isogenic control (**D**) and NINJ2-KO (**E**) MCF7 cells were treated with 20 μM of control peptide or NINJ2-A peptide for 24 h, and followed by mock-treatment or treatment with LPS (0.5 μg/mL) and Nigericin (10 μM) for 24 h. The cell viability was measured by CellTiter-Glo assay. * indicated *p* < 0.05 by student’s test. n.s, indicated no significance. (**F**,**G**) Isogenic control (**F**) and NINJ2-KO (**G**) Molt4 cells were treated with 20 μM of control peptide or NINJ2-A peptide for 24 h, and followed by mock-treatment or treatment with LPS (0.5 μg/mL) and Nigericin (10 μM) for 24 h. The cell viability was measured by CellTiter-Glo assay. * indicated *p* < 0.05 by student’s test. ns, indicated no significance.

Both NINJ1 and NINJ2 contain an N-terminal adhesion motif (NAM) that is required for homophilic adhesion [10,11,24]. We showed previously that a peptide derived from NINJ1 NAM (aa 26-37) suppresses cell growth [19]. Although the region from aa 1–30 in NINJ2 can inhibit cell adhesion [11], it is not certain whether such a peptide from NINJ2 NAM is capable of regulating cell growth. Thus, we generated a NINJ2 peptide spanning the region of aa 12-29, designated as NINJ2-A peptide (Figure 1A). Similarly, the NINJ1 peptide spanning the region of aa 26-37 from NINJ1 NAM, designated as NINJ1-A, was synthesized as a positive control (Figure 1A). Next, isogenic control, NINJ1-KO or NINJ2-KO MCF7 cells, which were previously generated [18,19], were used to determine whether NINJ1-A or NINJ2-A peptides inhibit cell viability measured by CellTiter-Glo assay. As expected, NINJ1-A peptide was able to inhibit cell viability and cooperated with LPS and Nigericin to further inhibit cell viability in isogenic control MCF7 cells (Figure 1B). Combined treatment of LPS and Nigericin is known to induce pyroptosis and subsequently, inhibit cell growth [25]. Interestingly, the loss of NINJ1 was able to abrogate the ability of NINJ1-A to inhibit cell viability regardless of LPS and Nigericin treatment in MCF7 cells (Figure 1C), consistent with our previous report [19]. Similarly, we found that NINJ2-A peptide also inhibited cell viability in isogenic control MCF7 cells but not NINJ2-KO MCF7 cells (Figure 1D,E, left panels). Moreover, NINJ2-A peptide cooperated with LPS and Nigericin to further inhibit cell viability in isogenic control MCF7 cells but not NINJ2-KO MCF7 cells (Figure 1D,E, right panels). To confirm the observations, isogenic control and NINJ2-KO Molt4 cells were used to measure the effect of NINJ2-A peptide on cell viability. Molt4 is a T lymphoblastic cell line [26] and the NINJ2-KO Molt4 cell line was generated and characterized previously [18]. Similarly, we found that cell viability was inhibited by treatment with NINJ2-A peptide and further inhibited by co-treatment of NINJ2-A with LPS and Nigericin in isogenic control but not NINJ2-KO Molt4 cells (Figure 1F,G). Together, these data indicate that NINJ1-A and NINJ2-A peptides inhibit cell viability, potentially via disruption of homophilic cell adhesion.

### 3.2. NINJ2-A Peptide Inhibits Cell Growth in a p53-Dependent Manner

**Figure 2:**
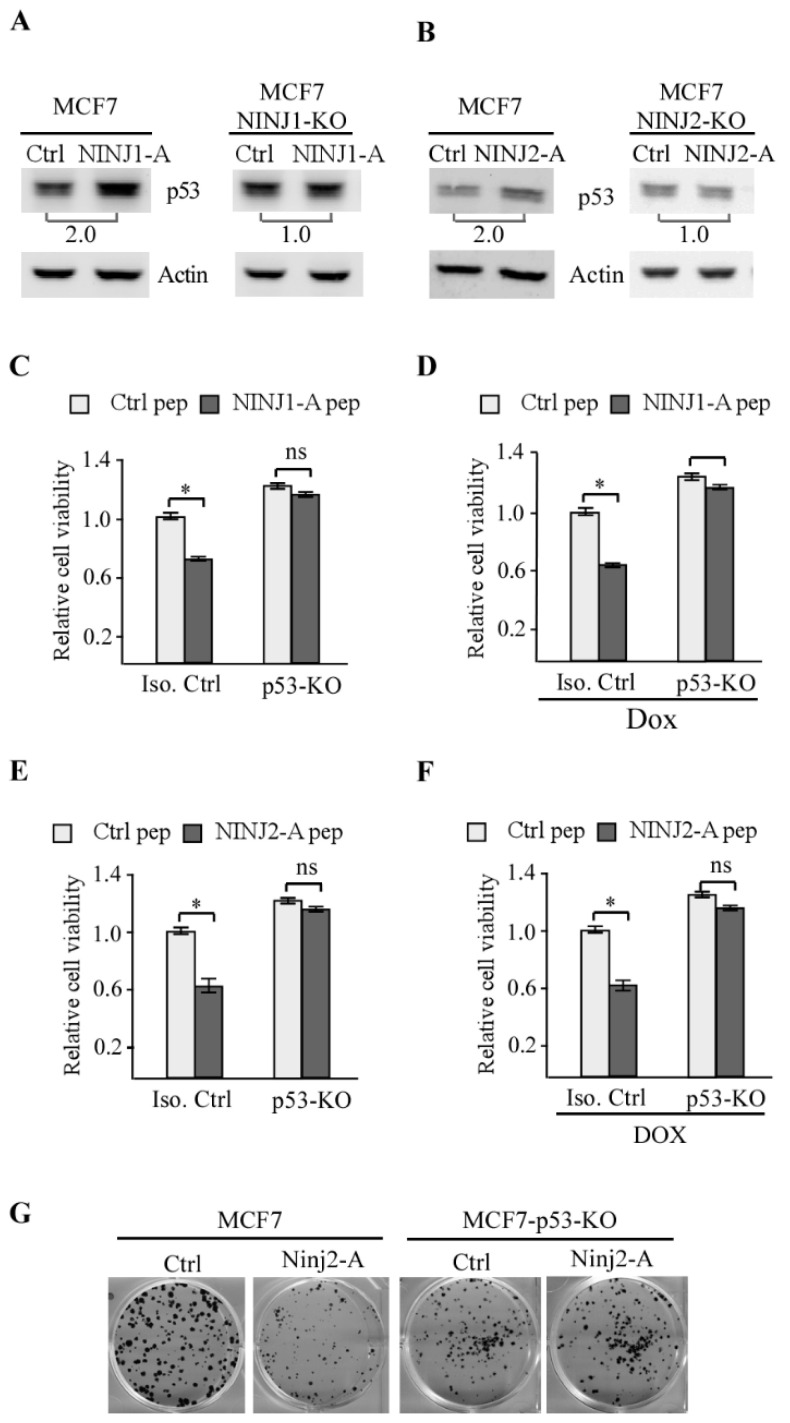
p53 is required for NINJ1 and NINJ2 Peptides-mediated growth inhibtion. (**A**) The level of p53 and actin protein was measured in isogenic control (left panel) and NINJ1-KO (right panel) cells treated with 20 μM of control peptide or NINJ1-A peptide for 24 h. The relative ratio of p53 protein (control peptide vs. NINJ1-A peptide) was shown below the lanes. (**B**) The level of p53 and actin protein was measured in isogenic control (left panel) and NINJ2-KO (right panel) cells treated with 20 μM of control peptide or NINJ2-A peptide for 24 h. The relative ratio of p53 protein (control peptide vs. NINJ2-A peptide) was shown below the lanes. (**C**,**D**) Isogenic control (**C**) and p53-KO (**E**) MCF7 cells were treated with 20 μM of control peptide or NINJ1-A peptide for 24 h, and followed by treatment with or without doxorubicin (100 μg/mL) for 12 h. The cell viability was measured by CellTiter-Glo assay. * indicated *p* < 0.05 by student’s test. ns, indicated no significance. (**E**,**F**) Isogenic control (**C**) and p53-KO (**E**) MCF7 cells were treated with 20 μM of control peptide or NINJ2-A peptide for 24 h, and followed by treatment with or without doxorubicin (100 μg/mL) for 12 h. The cell viability was measured by CellTiter-Glo assay. * indicated *p* < 0.05 by student’s test. ns, indicated no significance. (**G**) Colony formation was performed with Isogenic control and p53-KO MCF7 cells treated with a control peptide or NINJ2-A peptide (10 μM). The culture medium was replenished every three days over a period of two weeks.

We showed previously that like NINJ1, NINJ2 exerts a similar function in the p53 pathway as a target and a regulator of p53 [20,21]. In addition, we showed previously that NINJ1-A peptide inhibits cell growth by enhancing p53 expression [19]. Thus, we sought to determine whether NINJ2-A peptide activates the p53 signaling pathway. Upon treatment with NINJ1-A peptide, the level of p53 protein was increased in isogenic control but not in NINJ1-KO MCF7 cells (Figure 2A), consistent with previous reports [19]. Similarly, the NINJ2-A peptide was also able to induce p53 expression in isogenic control but not NINJ2-KO cells (Figure 2B). Next, to determine whether the enhanced expression of p53 by NINJ1-A or NINJ2-A peptide plays a role in growth suppression, cell viability was measured in isogenic control and p53-KO MCF7 cells. We found that cell viability was markedly inhibited by NINJ1-A (Figure 2C) and NINJ-2A (Figure 2D) in isogenic control MCF7 cells mock-treated or treated with doxorubicin, a DNA damage agent and known to induce p53 [27,28]. By contrast, both NINJ1-A and NINJ2-A peptides had very little effect on the cell viability in p53-KO MCF7 cells (Figure 2D,F). To verify that p53 is required for NINJ2-A peptide-mediated growth suppression, colony formation was performed. We found that NINJ2-A peptide markedly decreased the number of colonies in MCF7 cells but not in p53-KO MCF7 cells (Figure 2E), consistent with data from the cell viability assay (Figure 2E,F).

### 3.3. NINJ1 and NINJ2 Interact via Their N-Termini

**Figure 3:**
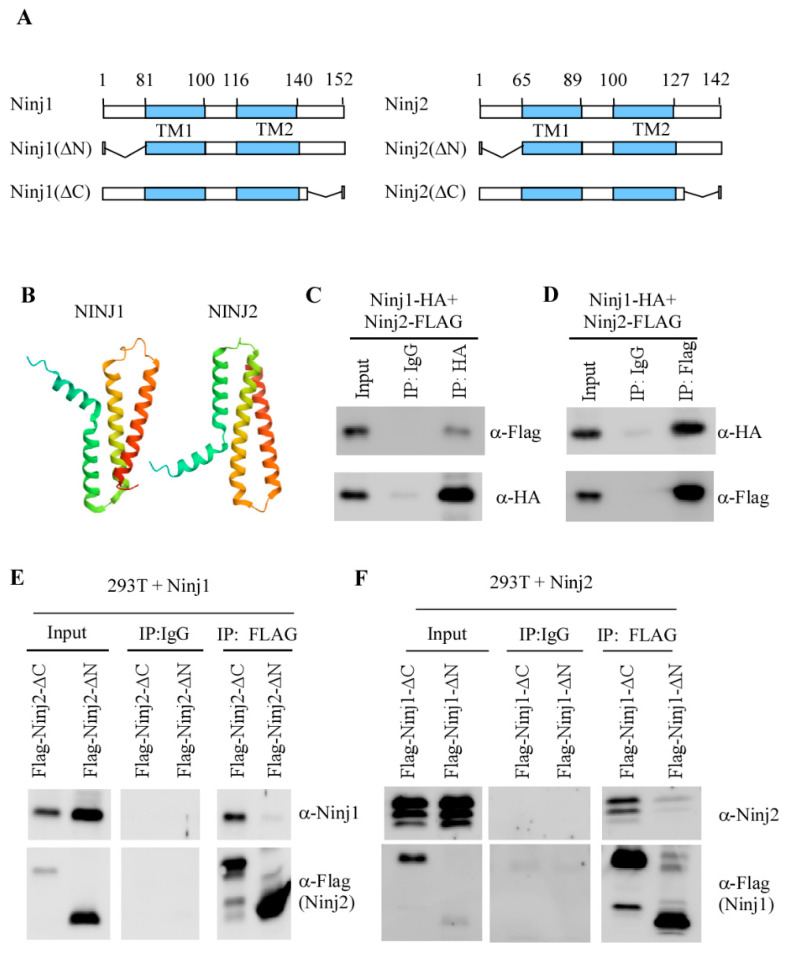
NINJ1 and NINJ2 associate via N-termini (**A**) Schematic representation of full-length NINJ1/2 proteins as well as various deletion mutants. (**B**) Represented Cryo-EM structure of NINJ1 (PDB ID: 8SZA) and NINJ2 (PDB ID: 8SZB) proteins. (**C**) MCF7 cells transfected with HA-tagged NINJ1 and Flag-tagged NINJ2 plasmids for 24 h. Cell lysates were collected and immunoprecipitated with control IgG or HA antibody, followed by Western blot to detect NINJ1 (α-HA) or NINJ2 (α-Flag). (**D**) The experiment was performed the same as in (**C**) excepted that Flag antibody was used for immunoprecipitation. (**E**) 293T cells were transfected with NINJ1 expression plasmid along with a plasmid expression N-terminal or C-terminal NINJ2 deletion mutant for 24 h. Cell lysates were collected and immunoprecipitated with control IgG or anti-Flag, followed by Western blot analysis to detect NINJ1 or NINJ2. (**F**) 293T cells were transfected with NINJ2 expression plasmid along with a plasmid expression N-terminal or C-terminal NINJ1 deletion mutant for 24 h. Cell lysates were collected and immunoprecipitated with control IgG or anti-Flag, followed by Western blot analysis to detect NINJ2 or NINJ1.

Like NINJ1, NINJ2 contains an N-terminal extracellular domain, a C-terminal extracellular domain and two transmembrane domains (Figure 3A). Recent CryoEM structural analysis showed that NINJ1 and NINJ2 have a similar 3D structure (Figure 3B) [29]. These data let us postulate whether NINJ1 and NINJ2 interact with each other since membrane proteins within the same family or superfamily often interact with each other to form functional complexes [30]. To test this, C-terminal HA-tagged NINJ1 and C-terminal Flag-tagged NINJ2 were transiently expressed in MCF7 cells, followed by IP–Western blot analysis. We found that Flag-tagged NINJ2 was detected in the NINJ1-immunocomplex but not in the IgG immunocomplex (Figure 3C). Similarly, HA-tagged NINJ1 was detected in the NINJ2-immunocomplex (Figure 3D). These data suggest that NINJ1 and NINJ2 interact with each other. Next, to map a region in NINJ1 or NINJ2 required for their association, two NINJ1 and 2 deletion mutants were generated (Figure 3A), which were then used for IP-Western blot analysis. We found that the full-length NINJ1 protein was able to associate with the NINJ2 C-terminal deletion mutant but not the N-terminal deletion mutant (Figure 3E), suggesting that the N-terminus of NINJ2 is required for NINJ1 to interact. Similarly, we found that the full-length NINJ2 protein was able to interact with the NINJ1 C-terminal deletion mutant but not the N-terminal deletion mutant (Figure 3F). Together, these data suggest that NINJ1 and NINJ2 interact with each other via their N-termini.

### 3.4. NINJ1-B and NINJ2-B Peptides, Which Are Derived from the N-Terminal Amphipathic Helices of NINJ1 and NINJ2, Respectively, Are Capable of Disrupting NINJ1-NINJ2 Interaction

**Figure 4:**
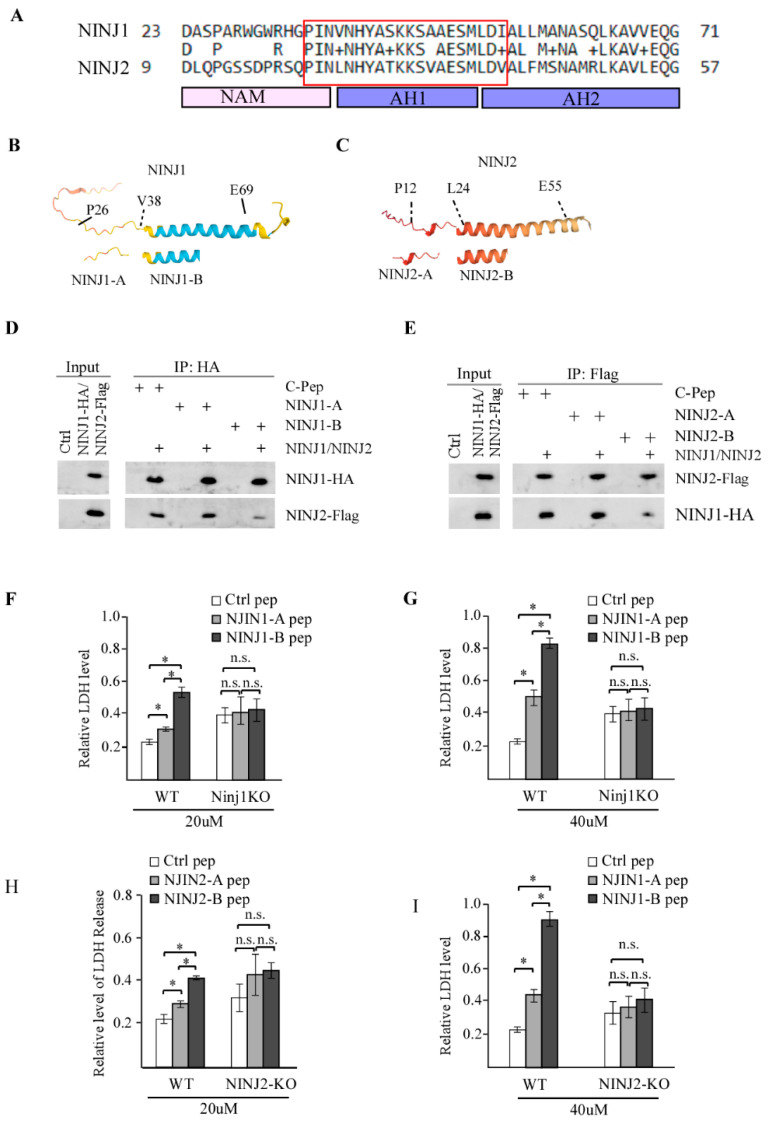
Identifying new peptides, NINJ1-B and NINJ2-B, disrupt the interaction between NINJ1 and NINJ2 (**A**) Sequence alignment of the N-terminus of NINJ1 and NINJ2 proteins. The location of NAM (N-terminal adhesion motif), AH1 (amphipathic Helix 1), and AH2 (amphipathic Helix 2) were shown below the sequence. The red box indicated the sequence of NINJ1-B and NINJ2-B peptides. (**B**,**C**) Schematic representation of the N-termini of NINJ1 and NINJ2 proteins and the location of NINJ1-A/B and NINJ2-A/B peptides. (**D**) 293T cells were transiently transfected with control pcDNA3 vector or HA-tagged NINJ1 and Flag-tagged NINJ2 vector for 24 h. Cell lysates were incubated with 20 μM of control peptide, NINJ1-A or NINJ1-B for 6 h, followed by immunoprecipitation with anti-HA. The immunocomplex was detected by anti-HA (NINJ1) or anti-Flag (NINJ2). (**E**) 293T cells were transiently transfected with control pcDNA3 vector or HA-tagged NINJ1 and Flag-tagged NINJ2 vector for 24 h. Cell lysates were incubated with 20 μM of control peptide, NINJ2-A or NINJ2-B for 6 h, followed by immunoprecipitation with anti-HA. The immunocomplex was detected by anti-HA (NINJ1) or anti-Flag (NINJ2). (**F**) Isogenic control and NINJ1-KO MCF7 cells were treated with 20 μM of control peptide, NINJ1-A or NINJ1-B peptide for 24 h and then treated with LPS (0.5 μg/mL) and Nigericin (10 μM) for another 24 h, followed by measurement of LDH release. * indicated *p* < 0.05 by student’s test. n.s, indicated no significance. (**G**) The experiment is performed the same as in (**F**) except that 40 μM peptide was used. * indicated *p* < 0.05 by student’s test. n.s, indicated no significance. (**H**) Isogenic control and NINJ2-KO MCF7 cells were treated with 20 μM of control peptide, NINJ2-A or NINJ2-B peptide for 24 h and then treated with LPS (0.5 μg/mL) and Nigericin (10 μM) for another 24 h, followed by measurement of LDH release. * indicated *p* < 0.05 by student’s test. n.s, indicated no significance. (**I**) The experiment is performed the same as in (**H**) except that 40 μM peptide was used. * indicated *p* < 0.05 by student’s test. n.s, indicated no significance.

The N-terminus of NINJ1 and NINJ2 consists of a flexible region and two amphipathic helices (Figure 4A). The flexible region, also known as NAM (N-terminal adhesion motif), is involved in cell adhesion while the two amphipathic helices are suggested to mediate the oligomerization of NINJ1 or NINJ2 [29]. Amino acid sequence alignment showed that the two amphipathic helices are very well conserved between NINJ1 and NINJ2 whereas the NAM motifs in NINJ1 and NINJ2 are less well conserved (Figure 4A). As amphipathic helices are known for protein–protein interactions [31,32], NINJ1 and NINJ2 may interact via their amphipathic helices. This observation prompts us to design a peptide from the amphipathic helix 1 (AH1) (Figure 4A, red box) that can disrupt the interaction between NINJ1 and NINJ2. The NINJ1-B peptide is derived from aa 35-54 in NINJ1 and the NINJ2-B peptide is derived from aa 21-40 in NINJ2 (Figure 4B,C). Next, we test whether NINJ1-B and NINJ2-B are able to interrupt NINJ1-NINJ2 interaction along with NINJ1-A and NINJ2-A peptides as controls. To this end, 293T cells expressing HA-tagged NINJ1 and Flag-tagged NINJ2 were treated with a control peptide, NINJ1-A, or NINJ1-B peptide followed by immunoprecipitation to detect the NINJ1 and NINJ2 interaction. We found that NINJ1 was able to bring down NINJ2 in the presence of a control peptide or NINJ1-A peptide (Figure 4D). By contrast, we found that in the presence of NINJ1-B peptide, the interaction between NINJ1 and NINJ2 was markedly attenuated (Figure 4D). Likewise, we found that NINJ2-B but not NINJ1-B peptide was able to disrupt the interaction between NINJ1 and NINJ2 (Figure 4E).

Since the loss of NINJ1 or NINJ2 leads to growth suppression [19,20,21], we reasoned that disruption of the NINJ1-NINJ2 complex would elicit similar effects on cell proliferation. To this end, isogenic control, NINJ1-KO and NINJ2-KO MCF7 cells were treated with a control peptide or various NINJ1/NINJ2 peptides along with LPS and Nigericin. LPS/Nigericin treatment is known to activate the NRLP3 inflammasome and subsequently induce pyroptosis [33]. Thus, the release of LDH, an indicator of cell death [34], was measured. We found that both NINJ1-A and NINJ1-B peptides were able to increase cell death in a dose-dependent manner in isogenic control MCF7 cells, which was abrogated by loss of NINJ1 (Figure 4F,G). Interestingly, the NINJ1-B peptide was much more potent than NINJ1-A in inducing MCF7 cell death at two different concentrations (Figure 4F,G). Similarly, we found that both NINJ2-A and NINJ2-B peptides were able to increase cell death in MCF7 cells in dose- and NINJ2-dependent manners (Figure 4H,I). Additionally, NINJ2-B peptide was much more potent than NINJ2-A peptide in inducing cell death (Figure 4H,I).

### 3.5. p53 Is Required for NINJ1-B and NINJ2-B Peptides to Induce Cell Death

**Figure 5:**
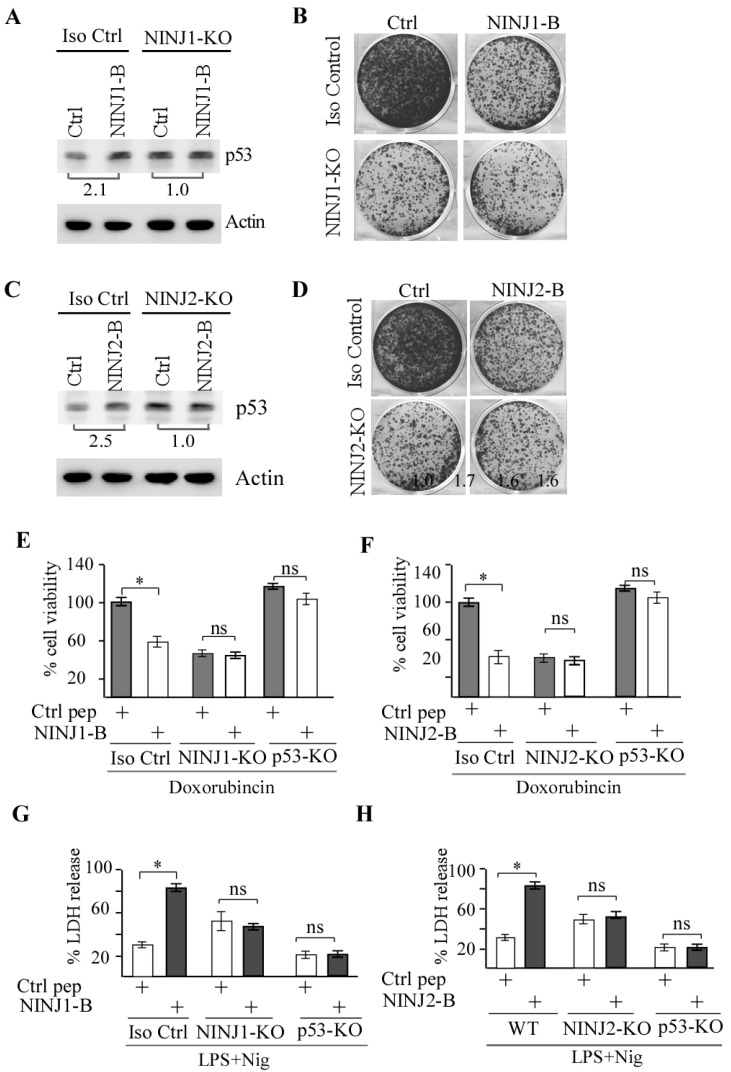
p53 is required for NINJ1-B and NINJ2-B peptide to inhibit cell proliferation (**A**) The level of p53 and actin protein was measured in isogenic control and NINJ1-KO MCF7 cells treated with 20 μM of control peptide or NINJ1-B peptide for 24 h. (**B**) Colony formation assay was performed with isogenic control and NINJ1-KO MCF7 cells treated with 5 μM of control peptide or NINJ1-B peptide. The culture medium was replenished every three days over a period of two weeks. (**C**) The level of p53 and actin protein was measured in isogenic control and NINJ1-KO MCF7 cells treated with 20 μM of control peptide or NINJ2-B peptide for 24 h. (**D**) Colony formation assay was performed with isogenic control and NINJ2-KO MCF7 cells treated with 5 μM of control peptide or NINJ2-B peptide with culture medium replenished every three days over a period of two weeks. (**E**) Cell viability assay was measured in isogenic control, NINJ1-KO, p53-KO MCF7 cells treated with 20 μM of control peptide or NINJ1-B peptide for 24 h, and then treated with or without doxorubicin (250 μg/mL) for 24 h. * indicated *p* < 0.05 by student’s test. ns, indicated no significance. (**F**) Cell viability assay was measured in isogenic control, NINJ2-KO, p53-KO MCF7 cells treated with 20 μM of control peptide or NINJ2-B peptide for 24 h, followed by treatment with or without doxorubicin (250 μg/mL) for 24 h. * indicated *p* < 0.05 by student’s test. ns, indicated no significance. (**G**) LDH release was measured in isogenic control, NINJ1-KO, p53-KO MCF7 cells treated with 20 μM of control peptide or NINJ1-B peptide for 24 h, and then combined treatment of LPS (0.5 μg/mL) and Nigericin (10 μM) for another 24 h. * indicated *p* < 0.05 by student’s test. ns, indicated no significance. (**H**) LDH release was measured in in isogenic control, NINJ2-KO, p53-KO MCF7 cells treated with 20 μM of control peptide or NINJ2-B peptide for 24 h, followed by combined treatment of LPS (0.5 μg/mL) and Nigericin (10 μM) for another 24 h. * indicated *p* < 0.05 by student’s test. ns, indicated no significance.

Previously, we showed that loss of NINJ1 or NINJ2 leads to increased p53 expression and growth suppression [20,21]. Thus, to gain a deeper understanding of the mechanisms through which NINJ1-B and NINJ2-B peptides suppress cell growth, we investigated whether p53 plays a role in the growth suppression mediated by these peptides. To this end, isogenic control and NINJ1-KO MCF7 cells were treated with a control peptide or NINJ1-B peptide and the level of p53 protein was measured by Western blot analysis. We found that the NINJ1-B peptide led to a marked increase in the level of p53 protein in isogenic control but not in NINJ1-KO MCF7 cells (Figure 5A, compare lanes 1 and 3 with 2 and 4, respectively), suggesting that NINJ1-B peptide enhances p53 expression in a NINJ1-dependent manner. We would like to note that knockout of NINJ1 led to elevated expression of p53 (Figure 5A, compare lane 1 with 3) as previously reported [19,20]. Next, colony formation assay was performed and showed that upon treatment of NINJ1-B peptide, the number of colonies was markedly decreased in isogenic control MCF7 cells (Figure 5B, left column), consistent with the observations that NINJ1-B peptide increases cell death induced by LPS and Nigericin treatment (Figure 4F,G). We also found that the increased expression of p53 by loss of NINJ1 inhibited colony formation (Figure 5B), which is consistent with previous reports [19,20]. However, the effect of NINJ1-B peptide on colony formation was much weaker in NINJ1-KO MCF7 cells than that in the isogenic control cells (Figure 5B).

To determine whether NINJ2-B peptide can induce p53 expression and inhibit colony formation assay, similar experiments were performed with isogenic control and NINJ2-KO MCF7 cells. We found that the NINJ2-B peptide was able to increase p53 expression (Figure 5C, compare lanes 1 and 3 with 2 and 4, respectively) and inhibit colony formation in isogenic control MCF7 cells (Figure 5D). By contrast, the NINJ2-B peptide had very little effect on p53 expression and growth suppression in NINJ2-KO MCF7 cells (Figure 5C,D). We would also like to note that knockout of NINJ2 led to elevated expression of p53 and inhibited colony formation (Figure 5C,D), consistent with previous reports [18,21].

Next, to verify the role of p53 in the growth suppression mediated by NINJ1-B or NINJ2-B peptide, p53-KO MCF7 cells were used. We found that the NINJ1-B peptide was able to markedly inhibit cell viability in MCF7 cells mock-treated or treated with doxorubicin (Figure 5E). By contrast, in p53-KO or NINJ1-KO MCF7 cells, NINJ1-B peptide was unable to reduce cell viability regardless of doxorubicin treatment (Figure 5E). Similarly, we found that NINJ2-B peptide reduced cell viability in MCF7 cells (Figure 5F). However, this inhibitory effect of the NINJ2-B peptide was not observed in p53-KO or NINJ2-KO MCF7 cells (Figure 5F). These data suggest that the mechanisms of action of NINJ1-B and NINJ2-B peptides require p53 and NINJ1/2 proteins. To verify these observations, the effect of NINJ1-B or NINJ2-B peptide on pyroptosis was assessed by measuring LDH release in the presence of LPS and Nigericin. We found that the NINJ1-B peptide was able to increase LDH release in MCF7 cells treated with LPS and Nigericin, and this effect was abolished in both p53-KO and NINJ1-KO MCF7 cells (Figure 5G). Similarly, upon treatment with NINJ2-B peptide, LDH release was also enhanced in MCF7 cells but not in p53-KO or NINJ2-KO MCF7 cells (Figure 5H).

## 4. Discussion

A short 10-50 aa peptide represents a promising frontier in cancer treatment due to its unique biochemical properties and versatility as it can selectively bind to cell surface receptors and proteins and act as either an agonist or an antagonist [35,36]. In the current study, we generated several peptides derived from the N-terminus of NINJ1 and NINJ2 and showed that peptides targeting cell adhesion or the NINJ1-NINJ2 complex can elicit growth suppression in breast cancer cells. We also found that NINJ1/2 peptides, which are derived from the amphipathic helix and capable of disrupting NINJ1-NINJ2 interaction, exhibit a greater potency in growth suppression. Furthermore, we showed that these NINJ1/2 peptides are a potent inducer of p53, which subsequently contributes to growth suppression. Our data shed light on the development of NINJ1/2 peptides as a potential cancer therapeutic agent, especially for tumors with wild-type p53.

Many cell adhesion molecules from the same family can interact with each other due to their structural similarities. In our study, we observed that NINJ1 and NINJ2 interact with each other via their N-termini (Figure 3). However, it is not clear whether NINJ1 and NINJ2 interact in cis, e.g., in the same membrane or in trans, e.g., on adjacent cells, which is worth further investigation. Moreover, it is not clear how NINJ1-NINJ2 interaction can modulate their activities. Several studies have shown that NINJ1 actively promotes lytic cell death by aggregating on the plasma membrane and directly causing its rupture [15,16,37]. Unlike NINJ1, NINJ2 can aggregate but not induce plasma membrane rupture, potentially due to its structure difference [18,29,37]. Considering these different roles of NINJ1 and NINJ2 in lytic cell death, it is possible that NINJ2 may interact with NINJ1 and subsequently, prevent NINJ1 from oligomerizing under normal conditions. These possibilities need to be carefully examined.

In this study, we found that NINJ1/2-B peptides, which target the NINJ1-NINJ2 interaction, were more potent in growth suppression than NINJ1/2-A peptides, which target the cell adhesion function of NINJ1/2 (Figure 4). These data suggest that the NINJ1 and NINJ2 interactions are critical for cell survival. Since both NINJ1 and NINJ2 are found to be over-expressed in several types of cancer, including breast cancer, targeting their interaction may be a feasible approach for cancer therapeutic development. Thus, further studies are warranted to optimize these NINJ1/2-B peptides. For example, the critical residue(s) required for NINJ1 and NINJ2 interaction should be identified, which would provide insight into how to shorten the peptide length. Incorporating D-amino acids or stapling the peptide may enhance their stability and efficacy. Moreover, as our study focused mostly on the first amphipathic helix of NINJ1/2, it would be interesting to determine whether peptides from the second amphipathic helix of NINJ1/2 can disrupt the interaction between NINJ1 and NINJ2 and subsequently inhibit cell growth.

Previously, we found that loss of NINJ1 or NINJ2 leads to enhanced p53 mRNA translation [21,24]. In this study, we found that p53 is induced by NINJ1/2-A and NINJ1/2-B peptides and shown to be responsible for subsequent growth suppression (Figure 2 and Figure 5). Thus, we postulate that both NINJ1/2-A and NINJ1/2-B peptides enhance p53 mRNA translation by blocking the NINJ1/2 signaling pathway. Thus, it is possible that these peptides can enhance the expression of mutant p53, which is known to function as an oncoprotein and thereby promote tumor growth. Thus, p53 status should be taken into consideration when developing such a therapeutic strategy. Furthermore, our peptides are still in the early stage of clinical development. To explore the therapeutic potential of these peptides, further studies are needed to characterize these peptides to enhance their tumor-killing efficacies. First, various biochemical analyses are needed to enhance the potency as well as efficacy of these peptides, e.g., measuring the binding affinities of NINJ1/2-A and NINJ1/2-B peptides to NINJ1 or NINJ2, increasing their stability via various chemical modifications, and optimizing the delivery methods. Second, animal studies are necessary to determine whether the NINJ1/2-A and NINJ1/2-B peptides can effectively inhibit tumor growth in a NINJ1/2- and p53-dependent manner in vivo. Third, pharmacokinetics studies are needed to determine how the peptides are absorbed, distributed, metabolized, and excreted by the body. By addressing these questions, we hope that the NINJ1/2-A or NINJ1/2-B peptides can be translated into clinical use.

## Data Availability

All study data are included within the article.
